# Statistical investigations of protein residue direct couplings

**DOI:** 10.1371/journal.pcbi.1006237

**Published:** 2018-12-31

**Authors:** Andrew F. Neuwald, Stephen F. Altschul

**Affiliations:** 1 Institute for Genome Sciences and Department of Biochemistry & Molecular Biology, University of Maryland School of Medicine, Baltimore, Maryland, United States of America; 2 Computational Biology Branch, National Center for Biotechnology Information, National Library of Medicine, National Institutes of Health, Bethesda, Maryland, United States of America; The Institute of Cancer Research, UNITED KINGDOM

## Abstract

Protein Direct Coupling Analysis (DCA), which predicts residue-residue contacts based on covarying positions within a multiple sequence alignment, has been remarkably effective. This suggests that there is more to learn from sequence correlations than is generally assumed, and calls for deeper investigations into DCA and perhaps into other types of correlations. Here we describe an approach that enables such investigations by measuring, as an estimated *p*-value, the statistical significance of the association between residue-residue covariance and structural interactions, either internal or homodimeric. Its application to thirty protein superfamilies confirms that direct coupling (DC) scores correlate with 3D pairwise contacts with very high significance. This method also permits quantitative assessment of the relative performance of alternative DCA methods, and of the degree to which they detect direct versus indirect couplings. We illustrate its use to assess, for a given protein, the biological relevance of alternative conformational states, to investigate the possible mechanistic implications of differences between these states, and to characterize subtle aspects of direct couplings. Our analysis indicates that direct pairwise correlations may be largely distinct from correlated patterns associated with functional specialization, and that the joint analysis of both types of correlations can yield greater power. Data, programs, and source code are freely available at http://evaldca.igs.umaryland.edu.

## Author summary

The success of Direct Coupling Analysis (DCA) for protein structure prediction suggests that multiple sequence alignments implicitly contain more structural information than had previously been realized, and prompts deeper investigations of the sequence correlations uncovered by either DCA or other approaches. To aid such investigations and thereby broaden the utility of and improve DCA, we describe an approach that measures the statistical significance of the association between DCA and either 3D structure or correlated patterns associated with functional specialization. This approach can be used to evaluate the relative performance of DCA methods, their ability to distinguish direct from indirect couplings, and the potential biological relevance and mechanistic implications of alternative conformations and homodimeric interactions.

This is a *PLOS Computational Biology* Methods paper.

## Introduction

Contacts among residues largely determine a protein’s three-dimensional structure. For proteins sharing a common structure, such contacts generally produce correlated substitution patterns between residue pairs. Over evolutionary time substitutions at one residue position often result in compensating substitutions at other positions in order to maintain critical interactions. This allows the prediction of protein structural contacts based upon multiple sequence alignment (MSA) covariance analysis. Early approaches were only partially successful, with a major shortcoming the confounding effect of indirect correlations: When residues at positions *i* and *j* correlate, as do those at positions *j* and *k*, then residues at positions *i* and *k* may also correlate even though they fail to interact directly. Direct Coupling Analysis (DCA) and related methods [1–8] have overcome this problem by disentangling direct correlations from indirect coupling effects. As used here, the term DCA refers to all such approaches. DCA constitutes a major breakthrough in protein structure prediction and is currently being applied successfully on a large scale [9].

DCA programs employ a variety of algorithmic strategies, including *sparse inverse covariance estimation* (PSICOV) [4], *pseudo-likelihood maximum entropy optimization* (EVcouplings-PLM) [5, 6] (CCMpred) [10] and *multivariate Gaussian modeling* (GaussDCA) [11]. DCA methods are evaluated by comparing those residue pairs with the highest direct coupling (DC) scores to residue-to-residue contacts within protein structures. Currently this involves using, for example, ROC curves [11], the Matthews correlation coefficient [12], F_1_ scores, or often the positive prediction value (PPV). Such measures are applied by labeling data points according to a binary classification scheme; for DCA, those residue pairs that are a specified distance apart within a benchmark structure (e.g., ≤ 5 Å) are labeled as positives and other pairs as negatives. However, there are reasons to criticize such measures in particular circumstances [13]. In particular, it is not clear how to assess the significance of such measures when comparing different proteins or distinct structures. To standardize such comparisons, it is desirable to obtain a measure of statistical significance, which also provides insight into how surprised we should be with a given result. As illustrated here, one can use such a measure to determine whether it is better to base DC scores on an MSA of more closely related proteins rather than on an entire superfamily MSA.

Given a set of structures for a protein superfamily, a significance measure can help identify those of greatest interest: Direct couplings between pairs of residues presumably are due to selective constraints maintaining functionally important structural interactions. Hence, those protein structures that exhibit the most biologically relevant interactions should achieve the highest level of significance. One could therefore use a significance measure to select among alternative structural models generated by homology or by *ab initio* structure prediction methods. One may also adapt such a measure to evaluate the degree to which high DC scores are associated with properties other than 3D structural contacts. As illustrated here, for example, one may determine whether those residues most distinctive of a particular protein family are overrepresented among the highest DC-scoring residue pairs.

Here we describe a method to estimate, in various contexts, the statistical significance of the correspondence between DC scores and either protein structural contacts or other protein properties. Unlike the current practice of selecting for analysis an arbitrary number of the highest scoring pairs (e.g., 1.5 times the MSA length [14]), our approach determines the optimal number of such pairs automatically based on a statistical criterion, while adjusting automatically for the number of multiple hypotheses tested. Unlike binary classification schemes, our approach takes into account both the order of each residue pair based on DC scores and their ranks based on 3D pairwise distances; hence, it treats the structurally closest residue pairs having high DC scores as of higher biological relevance than such pairs having low DC scores. By providing a quantitative measure of significance, our approach can detect subtle yet important features of the data that qualitative measures would fail to distinguish from background noise.

We illustrate this approach by investigating: the relative performance of alternative methods; the biological relevance of alternative structures; subtle structural changes associated with the transition state of Ran GTPase; the contribution of homo-oligomer interfaces to aggregate DC scores; DCA’s dependence on the sequences included in the input MSA; and the correspondence between DCA pairwise correlations and correlated patterns associated with protein functional specialization.

## Results

### Statistical models

Abstractly, given an array of elements ordered by a primary criterion (e.g., as used here, DC scores), we ask how well it agrees with a secondary criterion (e.g., 3D pairwise distances) that distinguishes and ranks a subset of the elements. More specifically, we seek to identify an optimal initial cluster of elements of the array (defined by a cut), as measured by a relevant *p*-value. Our approach is based upon Initial Cluster Analysis (ICA) [15]; see Methods. For reference, Table 1 provides a summary of the variables used below. ICA answers the question: Given a random array of length *L*, containing *D* '1's (representing distinguished elements), and *L*—*D* '0's, what initial cluster, consisting of elements up to and including a cut point *X*, contains the most surprising number *d* of '1's, and what is its probability of occurring? (Below, we call the *d* '1's in an initial cluster “left-distinguished elements.”) For *L* = 18 and *D* = 7, for example, one such array is “101101100000010001”, with optimal cut point *X* = 7 (underlined), yielding *d* = 5. Here we note that, in practice, to distinguish elements within our array, we frequently rank all the elements, and distinguish those with rank ≤ *D*. We then might denote our example array as “401603200000070005” with digits > 0 denoting the ranks of distinguished elements. ICA ignores these ranks when choosing the optimal *X*, whereas we would prefer the *d* distinguished elements to the left of *X* to have superior ranks (i.e., lower numbers) than those to the right.

**Table 1:** List of variables.

Symbol	Definition
*L*	Total number of column pairs in the ICA array
*r*	Maximum 3D distance used to define contacting residue pairs (default: 5 Å)
*D*	Number of contacting pairs, i.e. distinguished elements
*X*	Optimum cut point (as defined by the ICA algorithm) for partitioning an array of length *L*
*d*	Number of left-distinguished elements, i.e. contacting pairs to the left of the cut point *X* (inclusive)
*m*	Minimum sequence separation between residue pairs in a query protein of known structure
*P*_*a*_	Estimated *p*-value for finding *d* distinguished elements to the left of *X* in the array
*R*	The number, among the *d* elements with smallest pairwise distances, that occur to the left of *X* (used for calculating *P*_*b*_)
*P*_*b*_	The probability, based on the cumulative hypergeometric distribution, of *R* being at least the value observed
*P*_*J*_	Estimated joint *p*-value
*S*	-log_10_ *P*, where *P* corresponds to *P*_*J*_ after correcting for multiple tests
*x*	Constant cut point (used instead of an optimized cut point *X*)
ℓ	The length of the input MSA
*F*	Numerical factor defining the constant cut point as *x* = F × ℓ
*P*_*x*_	The probability, based on the cumulative hypergeometric distribution, of *d* being at least the number observed up to constant cut point *x*
*P*_*F*_	Estimated joint *p*-value that combines *P*_*b*_ and *P*_*x*_, where *x* = *F* × ℓ
*S*_*F*_	-log_10_ *P*_*F*_

To generalize ICA to exploit ranking information we incorporate a ball-in-urn model to calculate a ranking specific *p*-value *P*_*b*_. For a specific cut point *X* that yields *d* left-distinguished elements, we imagine first coloring red, among all *D* distinguished elements, those *d* elements with superior ranks (e.g., with the smallest pairwise distances); and then recording the number *R* that are red among the left-distinguished elements. Ideally, all the left-distinguished elements will outrank the remaining distinguished elements, yielding *R* = *d*, but more generally higher values of *R* are better; in the example of the previous paragraph, *D* = 7, *d* = 5 and *R* = 4. Given the null hypothesis that rankings are random, we may then use the cumulative hypergeometric distribution to calculate the probability *P*_*b*_ that ≥ *R* of the left-distinguished elements are red:

$$P_{b}=\left[\sum_{i=R}^{d}\left(\begin{array}{c} d\\ i \end{array}\right)\left(\begin{array}{c} D-d\\ d-i \end{array}\right)\right]÷\left(\begin{array}{c} D\\ d \end{array}\right).$$

This corresponds to drawing *d* balls from an urn containing *D* balls, of which *d* are red; note that the number of balls drawn here equals the number colored red. A low value of *P*_*b*_ is reported for a cut with a surprising number, among its *d* left-distinguished elements, having the *d* smallest pairwise distances.

Before it corrects for optimizing over all possible cuts, ICA can be understood as calculating a *p*-value *P*_*a*_ for finding *d* distinguished elements to the left of a cut point *X*. Because the calculation of *P*_*a*_ ignores ranking information, it will be independent of *P*_*b*_, and these two *p*-values may therefore be combined to yield a joint *p*-value *P*_*J*_ [16–19] using the formula

$$P_{J}=P_{a}P_{b}\left(1-\ln P_{a}P_{b}\right).$$

Low values of *P*_*J*_ may arise from low values of *P*_*a*_, or *P*_*b*_, or of both. *P*_*J*_ can provide a statistically stronger measure than *P*_*a*_ alone of the congruence of two orderings, here derived from DC scores and 3D distances. The *p*-values *P* we report in this paper correspond to *P*_*J*_, after it has been corrected for optimization over the multiple cut points *X* considered, as described in [15]. One may wish to optimize as well over various values of *D*, but in the current application larger values of *D* are then almost always preferred, due to the indirect couplings considered below. We therefore choose a fixed *D*, based upon a maximum allowed 3D distance within a reference structure.

To summarize, in order to apply the theory above to the question of how well DCA actually uncovers direct contacts within proteins, we proceed as follows. Given an MSA, a method to calculate DC scores for all column pairs, and a reference structure corresponding to one of the sequences in the MSA, we consider only those pairs of MSA columns separated by ≥ *m* intervening positions within the reference sequence, with *m* = 5 by default. Ordering these column pairs by descending DC score yields our array of elements, of length *L*. We then distinguish those *D* elements whose 3D distance per the reference structure is ≤ *r* Å, with *r* = 5 by default, and rank them by increasing distance. (This distance is defined as the minimum between sidechain atoms, including hydrogens, of the paired residues. For glycine, C_α_ and its attached hydrogen serve as the sidechain atoms.) ICA's original *P*_*a*_ depends only upon the specification of these pairs as distinguished, whereas *P*_*J*_ takes account as well of their rankings, through the ball-in-urn derived *P*_*b*_. As we will show below, for this application *P*_*a*_ is, in general, far smaller than *P*_*b*_. However, we have found *P*_*J*_ to provide, in general, greater statistical power than *P*_*a*_ for analyzing protein sequence-structural relationships, and our focus in this paper is to illustrate its use.

### Defining *P*_*x*_ and *P*_*F*_

Currently DCA performance is often evaluated using the positive prediction value (PPV), defined as the percentage of observed reference-structure 3D contacts corresponding to a fixed number *x* (e.g., *x* = 100 [11, 20]) of the highest DC-scoring column pairs. In contrast, the cut point *X* is not fixed but chosen to optimized significance. Because the number of column pairs grows with increasing MSA length ℓ, *x* is often chosen, using a parameter *F*, as *x* = *F* × ℓ. Typical values of *F* range from 0.5 to 1.5 [10, 14]. Since we propose the *S*-score as a replacement for PPV, we compare these two metrics below in several ways. To aid these comparisons, we define *P*_*x*_ as the probability, based on the cumulative hypergeometric distribution, of *d* being at least the value observed for a constant value of *x* = *F* × ℓ. We define *P*_*F*_ as the estimated joint *p*-value that combines *P*_*b*_ and *P*_*x*_:

$$P_{F}=P_{x}P_{b}\left(1-\ln P_{x}P_{b}\right)=P_{F\times l}P_{b}\left(1-\ln P_{F\times l}P_{b}\right).$$

*Implementation and availability*. We implemented these algorithms and statistical models in C++ as the STARC (Statistical Tool for Analysis of Residue Couplings) program, which, along with the source code, is freely available at http://evaldca.igs.umaryland.edu.

### Simulations

**Fig 1:**
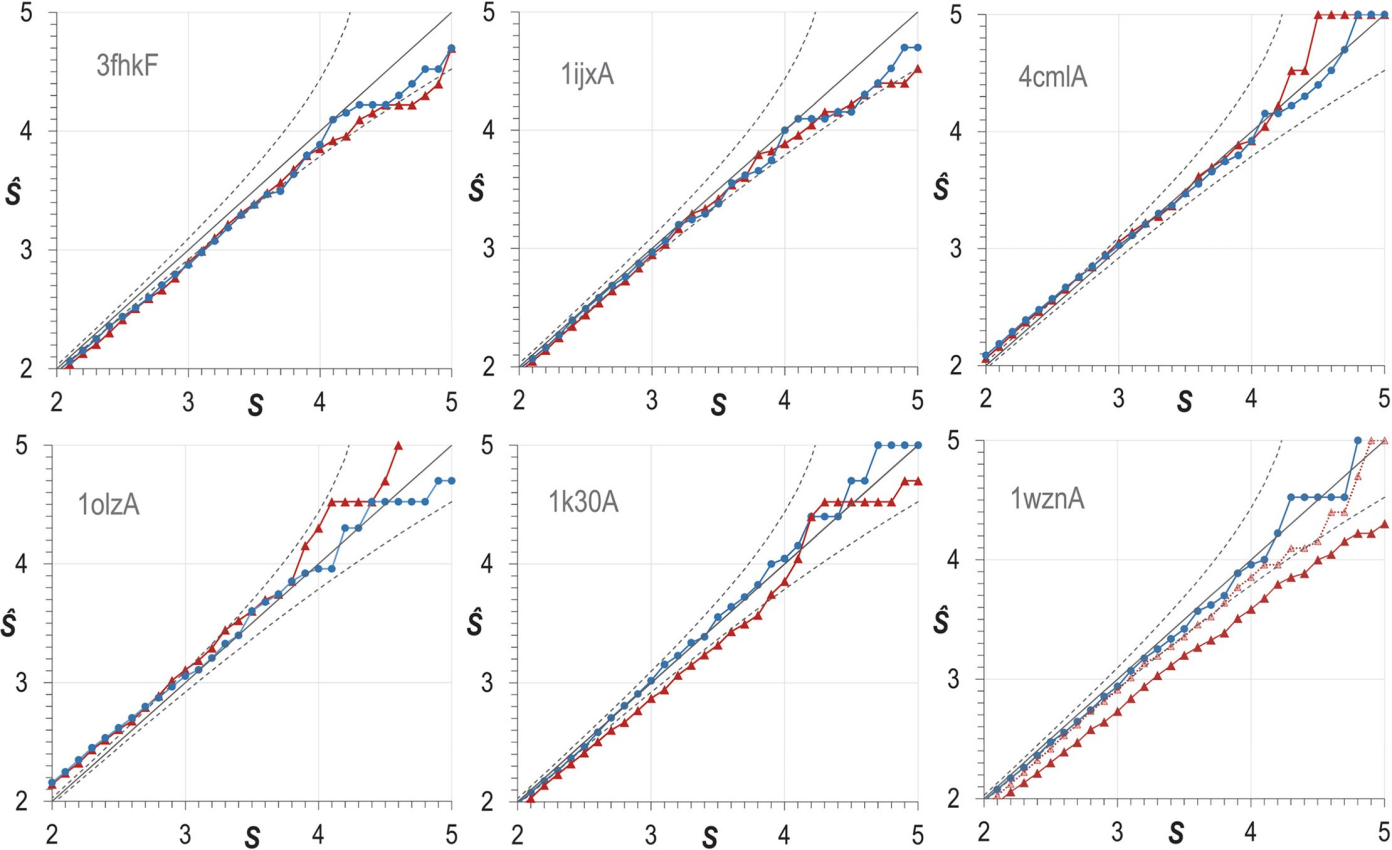
Empirical values of *Ŝ* as a function of *S* yielded by randomly shuffled 100,000 DCA arrays (blue dots connected by lines), and by 100,000 DCA arrays derived from column-permuted MSAs, where the order of the columns and of the residues within each column were randomly permuted (red triangles connected by lines). Solid straight lines represents agreement of *Ŝ* with *S*, and the dashed curves represent an error range of two standard deviations. Results are shown for six of the domains listed in Table 2, designated by their corresponding pdb identifiers 3fhkF, 1ijxA, 4cmlA, 1olzA, 1k30A, 1wznA, ordered by increasing numbers of sequences in their corresponding MSAs. For 1wxnA, the additional data points (faint red triangles connected by a dashed line) corresponds to an MSA of 5,117 sequences randomly drawn from the original MSA.

Here and below, for an estimated or theoretical *p*-value *P* we define a corresponding *s*-score as *S* = −log_10_ *P*. Our theory should yield accurate *p*-values and *s*-scores for randomly generated, or shuffled arrays. However, in the present application many column pairs within an MSA are interrelated (e.g., {*i*,*j*}, {*j*,*k*} and {*i*,*k*}), possibly affecting their DC scores as well as the corresponding distances derived from a structure. To test whether computed *p*-values remain valid given such interrelationships we generated, based on randomization of each of six MSAs with six corresponding structures, sets of random *p*-values, as described in Methods. We define *Ŝ*, as a function of *S*, to be -log_10_ of the proportion of *observed* (simulation-based) *s*-scores that are greater than or equal to *S*. If our *p*-value calculations are accurate, *Ŝ* should equal *S* to within stochastic error. In **Fig 1** we plot, for *S* from 2 to 5, the *Ŝ* obtained from 100,000 *p*-values for MSAs in which the residues in each column and the order of the columns for each MSA were randomly permuted (termed column-permuted MSAs). This operation retains the distribution of column relative entropies observed in the original MSA. For comparison, we plot as well the *Ŝ* obtained from an equivalent number of shuffled DC arrays (see Methods). These arrays abolish interrelationships among DC-scores, and so conform better to theory. The straight, solid line represents the agreement of *Ŝ* with theory, and dashed curves represent error ranges of two standard deviations. As can be seen, within stochastic error, *Ŝ* agrees well with theory for the shuffled arrays. (Because we can generate *p*-values rapidly for shuffled arrays, we have confirmed the accuracy of *Ŝ* in this case for *S* ≤ 8.)

For the MSA with the largest number of sequences (146,217) among the six used for column-permuted simulations, and corresponding to structure 1wznA, the values of *Ŝ* deviate consistently below the error bounds corresponding to slightly inflated *s*-scores. When we randomly removed all but 5,117 (3.5%) of the sequences in this MSA, however, this effect was essentially eliminated (see 1wznA plot in Fig 1). Values of *Ŝ* for the other column-permuted MSA simulations exhibit less of a tendency to deviate outside of the error bounds. Based on these examples, it appears that for large alignments, *s*-scores may be slightly inflated, but it is not clear why this is so.

We cannot be sure that values of *Ŝ* for randomized MSAs will conform to theory beyond the range tested. However, we may apply *s*-scores in a manner similar to that of *Z*-values. A *Z*-value is the distance between a raw score and the population mean in units of standard deviation. One may convert a *Z*-value into a *p*-value under the assumption (based on the Central Limit Theorem) that the variables are drawn from a normal distribution. Although this assumption is typically invalid for raw scores far away from the mean, *Z*-values still provide a useful metric for assessing significance. Extreme *s*-scores likewise provide a useful measure of statistical significance even though the true distribution may depart from the theoretical distribution used here.

### Application: Comparisons among DCA methods

We ran the STARC program on the output from four DCA programs, EVcouplings (EVC) [5, 6], GaussDCA with Frobenius norm ranking (GSF) [11], PSICOV (PCV) [4], and CCMpred (CCM) [10], each applied to thirty protein domain MSAs with reference 3D contacts ≤ 5 Å (**Table 2**). For a given MSA, better performing programs should typically generate more significant results, and thus generally higher *s*-scores.

**Table 2:** *S* calculated with and without *P*_*b*_ for thirty superfamilies using residue pairwise 3D distances ≤ 5 Å and a minimum of 5 intervening residues.

Query	Resolution	Query	Description	Number of	*S* ^a^				*S* without *P*_*b*_			
	(Å)	length		sequences	CCM	EVC	GSF	PCV^b^	CCM	EVC	GSF	PCV
1ayaA	2.05	101	tyrosine phosphatase SH2 domain	12,208	55	54	**60**	43	56	55	**62**	44
1b5oA	2.20	382	aspartate aminotransferase	105,741	**862**	810	774	606	**860**	805	768	601
1el3A	1.70	315	aldose reductase	67,824	**612**	588	514	502	**607**	582	509	499
1k30A	1.90	234	glycerol-3-phosphate acyltransferase	12,225	110	**114**	90	77	110	**112**	90	78
1b23P	2.60	184	elongation factor Tu	78,839	**167**	135	109	101	**167**	134	109	102
1olzA	2.0	481	Sema4D	5,453	187	**245**	185	92	184	**240**	183	91
1wznA	1.90	155	SAM-dependent methyltransferase	146,217	**169**	156	159	154	**164**	152	155	149
1z0kC	1.92	164	Rab4 GTPase	64,211	**212**	181	202	178	**209**	180	201	175
1zp9A	2.00	258	Rio1 serine kinase	24,076	105	**110**	91	86	106	**111**	92	87
2b61A	1.65	357	homoserine transacetylase	47,508	**294**	290	284	274	**294**	290	284	275
3ex7H	2.30	241	DEAD-box ATPase eIF4AIII	98,478	**254**	239	173	157	**251**	237	173	156
4ag9A	1.76	165	glucosamine-6-phosphate acetylase	107,738	167	**174**	173	163	164	**171**	170	163
5dfiA	1.63	318	apurinic-apyrimidinic endonuclease	36,297	293	**317**	244	193	291	**313**	242	193
5hf7A	1.54	227	thymine DNA glycosylase	7,588	125	**126**	72	66	**126**	125	73	66
5m4pA	2.30	164	pyruvate dehydrogenase kinase	1,651	33	34	**37**	23	34	36	**38**	24
1ijxA	1.90	127	cysteine-rich domain of sFRP-3	3,224	24	**25**	19	20	**25**	25	20	21
2nrlA	0.91	147	myoglobin	9,514	**98**	95	68	58	**97**	94	69	58
4cmlA	2.30	313	INPP5B	4,724	244	247	**266**	177	242	242	**264**	176
1jw9B	1.70	249	molybdopterin synthase MoeB	23,170	**331**	318	272	243	**325**	312	269	242
3fhkF	2.30	147	disulfide isomerase	1,042	61	64	**67**	49	61	64	**68**	49
3h7uA	1.25	335	plant stress-response enzyme Akr4c9	67,652	**589**	573	502	481	**577**	565	494	473
1g9rA	2.00	311	galactosyltransferase LgtC	10,575	**283**	264	281	212	**275**	254	274	208
4em8A	1.95	148	ribose 5-phosphate isomerase B	7,217	**184**	181	160	146	173	**175**	153	138
1i6mA	1.72	328	tryptophanyl-tRNA synthetase	20,731	**321**	312	198	166	**316**	309	194	165
3f1lA	0.95	252	oxidoreductase, Ycik	99,991	**454**	448	433	397	**446**	439	426	393
1jr3A	2.2	373	bacterial DNA clamp loader γ subunit	24,739	**377**	373	258	245	**373**	365	258	245
1nnlA	1.53	225	human phosphoserine phosphatase	130,332	**136**	133	111	91	**137**	131	111	93
1frwA	1.75	194	E. coli MobA	79,445	193	179	**213**	168	194	180	**212**	169
1bqbA	1.72	301	Aureolysin metalloproteinase	5,289	333	**336**	254	190	325	**331**	252	190
2ovdA	1.8	182	human complement protein C8γ	6,874	62	**75**	53	42	63	**75**	53	41
				average:	**245**	240	211	180	**242**	237	209	179

For each query the optimal *S* among competing methods is shown in bold. Shaded scores indicate the query for which the optimal method changes when *P*_*b*_ is excluded.

^a^Hydrogen atoms were added using the Reduce program [21], except for 3f1lA for which hydrogens were already present in the pdb coordinate file.

^b^PSICOV version 2.4 using the recommended –p and –d 0.03 options.

#### Optimal cut points and contact predictions

The *s-score*s reported in Table 2 confirm that DC scores correlate with 3D pairwise contacts with very high significance: for most DCA methods, *S* is > 200 on average. Table 2 also shows the corresponding *S*-scores computed without the *P*_*b*_ component. Omitting this component changes the *S* scores, on average, by 1–3 units, corresponding to changes of 1–3 orders of magnitude in *p*-values. In only three cases this changed the rankings between methods, all three of which had similar *S*-scores. Therefore, including the *P*_*b*_ component provides significant additional information without substantially influencing comparisons among methods, which we explore in the next section. (For the complete set of data for Table 2, including values of *L*, *X*, *D*, *d* and *R*, see S1 dataset and S2 dataset.)

#### Comparisons among DCA methods

To evaluate the relative performance of various DCA methods we applied to the data in Table 2 the two-tailed Wilcoxon signed-rank test [22], which is a non-parametric statistical hypothesis test for comparing two matched samples. We used this test to determine whether there is a significant tendency for one DCA method’s *s*-scores to be higher than those of another DCA method. We first normalized each *s*-score through division by the total number of residue pairs for its input MSA; the resulting normalized *s*-scores approximately follow a Gaussian distribution (see Methods). Since this test is based on thirty pairs of *s*-scores, the sum of the Wilcoxon signed ranks tend to follow a Gaussian distribution. The Wilcoxon test returns a *Z*-value for each pair of methods and a corresponding two-tailed *p*-value (**Table 3**). For the S-score, this test ranked CCMpred as performing only marginally better than EVcouplings (*p* = 0.09); EVcouplings significantly better than GaussDCA (*p* = 0.001); and GaussDCA significantly better than PSICOV (*p* = 2×10^−6^). For individual MSAs, the contribution of *P*_*b*_ to *P*_*J*_ varied, for CCMpred, from insignificant to highly significant (e.g., *P*_*b*_ = 6.3×10^−17^ for 3h7uA) with a geometric mean of *P*_*b*_ = 4.6×10^−7^, but the exclusion of *P*_*b*_ did not substantially affect the Wilcoxon test *p*-values comparing the methods. The superior performance of both CCMpred and EV-couplings is not surprising, as both are based on pseudo-likelihood maximization (PLM), which was first introduced as GREMLIN [23] and which was later shown [24, 25] to be more accurate than newer, faster methods such as PSICOV [4].

**Table 3:** Wilcoxon Signed Rank 2-tailed tests for the 30 analyses in Table 2.

Comparison		*S*-score		*S* without *P*_*b*_		*S*_*F* = 1.5_		*S*_*F* = 1.0_	
method 1	method 2	Z-value	*p*-value^a^	Z-value	*p*-value	Z-value	*p*-value	Z-value	*p*-value
CCM	EVC	1.70	9×10^−2^	1.82	7×10^−2^	2.76	6×10^−3^	4.00	6×10^−5^
CCM	GSF	3.71	2×10^−4^	3.67	2×10^−4^	3.98	7×10^−5^	4.56	5×10^−6^
CCM	PCV	4.78	2×10^−6^	4.78	2×10^−6^	4.70	3×10^−6^	4.72	2×10^−6^
EVC	GSF	3.22	1×10^−3^	3.16	2×10^−3^	3.28	1×10^−3^	3.10	2×10^−3^
EVC	PCV	4.76	2×10^−6^	4.78	2×10^−6^	4.47	8×10^−6^	4.10	4×10^−5^
GSF	PCV	4.76	2×10^−6^	4.70	3×10^−6^	3.53	4×10^−4^	2.34	2×10^−2^

^a^Note that *p*-value estimates below ~10^−4^ are unreliable.

#### Indirect couplings

Ideally, as their name indicates, DC scores should correspond to *direct* correlations between pairs of columns in an MSA. However, if a DCA method generates output inconsistent with this assumption, by picking up indirect couplings, our approach may yield significant *p*-values (i.e., high *S*) arising from pairs of residues distant in the 3D structure. Ideally, in the absence of indirect couplings, DC scores corresponding to distant pairs alone should not be significant. Note, however, that high *S* for large distances may be due in part to pairs directly coupled in an alternative conformation, or indirectly coupled via functional interactions mediated by other molecules or by a homo-oligomeric interface. Indirect couplings may also be due to phylogenetic correlations among closely related proteins.

**Fig 2:**
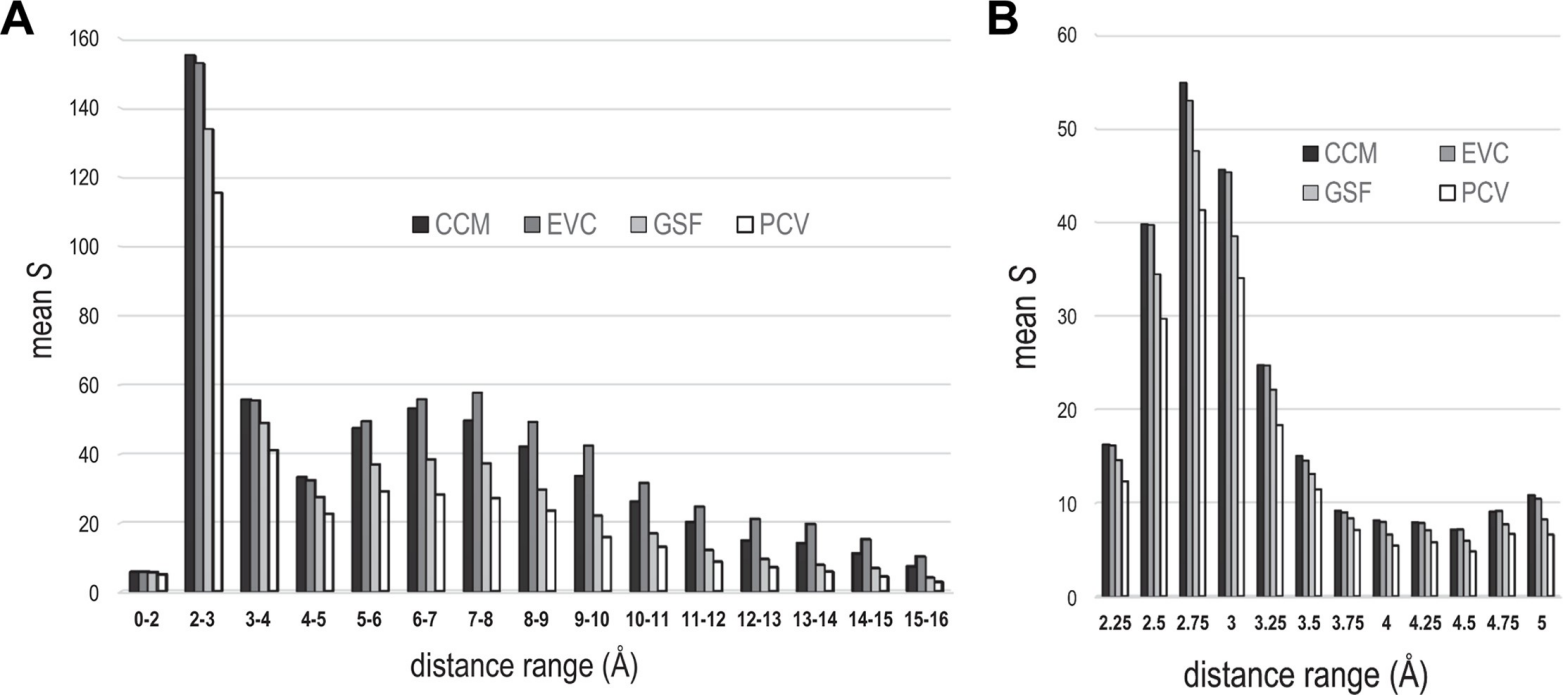
*S* as a function of 3D distance ranges defining distinguished residue pairs. See discussion in text. **A**. The *s*-scores obtained for distance ranges spanning zero to 16 Å. Column pairs corresponding to residue-to-residue distances below the indicated range were excluded from the analysis. **B**. Detailed plot of the span 2 to 5 Å. Each distance range covers 0.25 Å and is labeled by its upper limit.

In **Fig 2** we present bar plots for *S*, averaged over the thirty superfamilies of Table 2, based on various distance ranges used to define residue pairs as discriminating. (Note that we discarded from the DCA array all pairs corresponding to 3D distances below each specified range.) The high values of *S* we obtained for distant pairs suggests that all four methods are detecting couplings well beyond a residue-to-residue distance of 5 Å—EVcouplings more so than the other methods. For example, in the 2–3 Å range, *S* for CCMpred is significantly higher on average than for EVcouplings (*Z*-value = 3.57; *p* = 4×10^−4^), but in the 7–8 Å and 9–10 Å ranges, *S* for EVcouplings is significantly higher (*Z* = 2.89, *p* = 0.004 and *Z* = 3.24, *p* = 0.001, respectively).

#### *S*- and *S*_*F*_-scores versus PPV

To properly compare our scoring approach with PPV and to apply it to a fixed number of the highest DC-scoring pairs, rather than as a global metric based on all pairs, we define the alternative *s*-score *S*_*F*_ = −log_10_ *P*_*F*_. We computed *S*_*F* = 1.5_ and *S*_*F* = 1.0_ scores for the proteins in Table 2 (S2 dataset). These analyses retain the same Wilcoxon rankings as for *S* (Table 3), though at different levels of significance. *S*-scores are optimized over values of *X* and thus of *F*. For the 30 analyses in Table 2, the median value of *F* was 3.2 with a range of 0.6 to 18.4 (S1 dataset). Thus, the optimized *F* tends to be higher than conventional fixed values of *F* based on *ad hoc* criteria. An optimized *F* = 18.4 was obtained for Sema4D (pdbid: 1olzA), which is a large, irregularly shaped domain containing seven β-propeller structural repeats and for which artifactual correlations between repeats may cause DCA to mis-assign residue pairs leading to a high *F*. This elevated *F* also may be due, in part, to the tendency for large, elongated domains to have a lower percentage of internal contacts (1.1% in this case).

**Fig 3:**
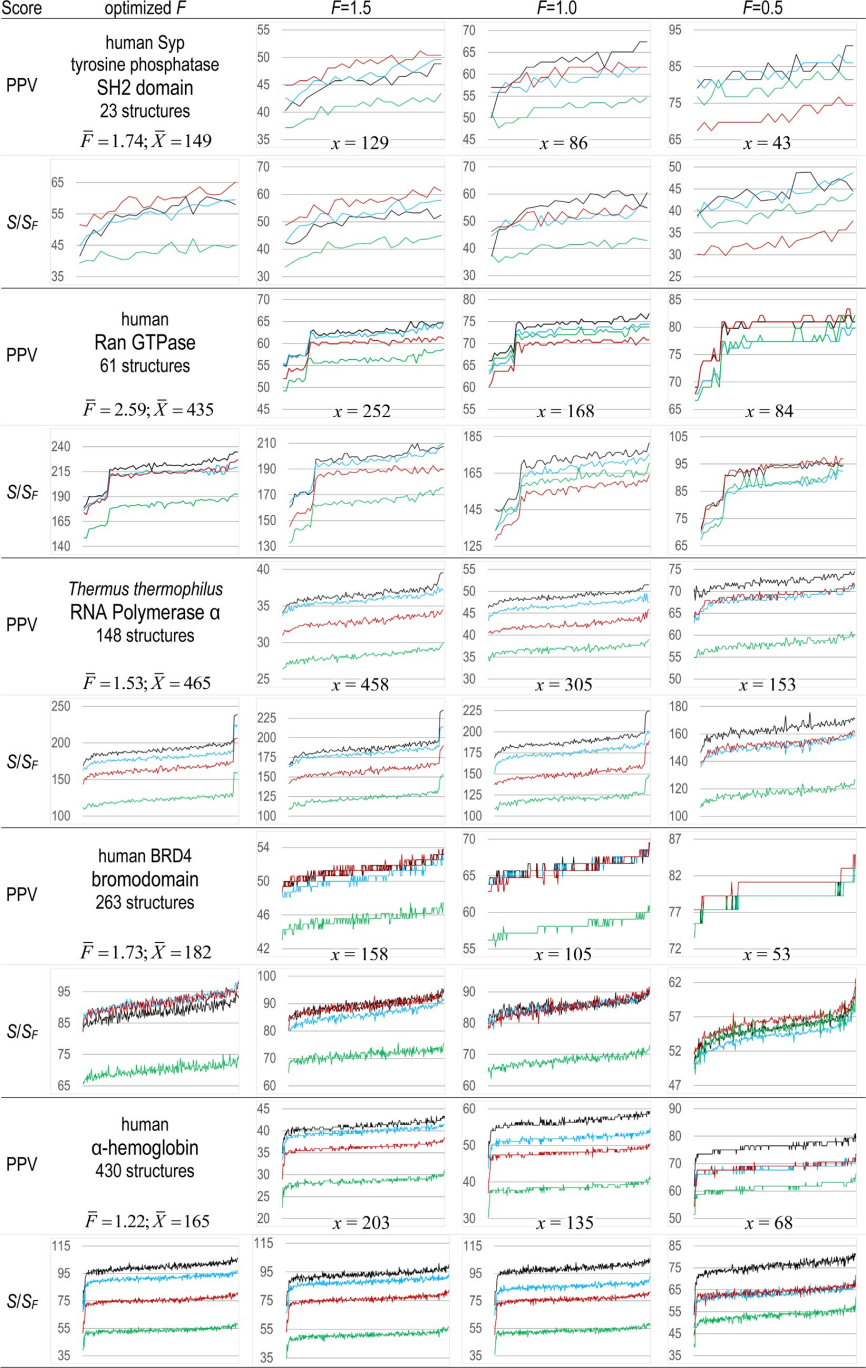
*S*, *S*_*F*_ and PPV scores as a function of various 3D structural coordinates for each of five protein domains. Structures are ordered by the average of their scores over four methods: CCM (black lines), EVC (cyan lines), GSF (red lines) and PCV (green lines). Below the name for each domain are shown both the mean value of *F* and of the optimal cut points *X* for the *S*-scores. The constant cut point values of *x* = *F* × ℓ are shown between the PPV and *S*_*F*_ plots. The value of *r* (the maximum 3D distance defining contacting pairs) is 5 Å.

Fig 3 shows, for each of five protein domains, how *S*, *S*_*F*_ and PPV depend on a range of structures for each protein, where *F* = 1.5, 1.0 or 0.5 for *S*_*F*_ and PPV. These include domains in four human proteins: the SH2 domain of Syp tyrosine phosphatase, Ran GTPase, the bromo domain of BRD4, and α-hemoglobin; and one bacterial protein: *Thermus thermophilus* RNA Polymerase α. Ran and Syp SH2 appear to span a broader range of conformational states than do the other proteins, which may explain the high variability in their *S*, *S*_*F*_ and PPV scores. For a given *F*, ranking of the four methods using *S*_*F*_ is similar to ranking them using PPV. For some proteins, however, when using either *S*_*F*_ or PPV the ranking of methods changes among values of *F*. For the Syp SH2 domain, GSF ranks 1^st^, 2^nd^ and 4^th^ for *F* = 1.5, 1.0 and 0.5, respectively. In contrast, for Ran GTPase, GSF ranks 3^rd^, 4^th^ and tied for 1^st^ for *F* = 1.5, 1.0 and 0.5, respectively. Hence, it seems unlikely that any one method will be consistently preferred for either small or large *F*. For each of the five analyses, Fig 3 shows the mean values of *X* and of *F* for the *S*-score plots and the values of *x* for *F* = 1.5, 1.0 and 0.5. Giving each of the five analyses equal weight, for S-scores the mean value of *F* equals 1.76.

For the α subunit of *T*. *thermophilus* RNA polymerase, *S*, *S*_*F* = 1.5_ and *S*_*F* = 1.0_ scores computed using any of the methods tested are considerably higher for a crystal structure of the class II transcription activation complex (pdb_id: 5i2d) [26] than for other structures: the right-most spikes in the Fig 3 plots for this protein correspond to these elevated scores. A similar spike in the PPV score for this structure is clearly evident only for the CCM method with *F* = 1.5. This complex consists of two α subunits and six other protein subunits bound to promoter DNA and a ribotetranucleotide primer and thus is likely to be more relevant biologically than other structures of this protein. This suggests that *S* and *S*_*F*_ scoring may be useful for assessing the biological relevance of structural conformations. In the next section, we further investigate using *S*-scores in this way.

### Application: Quantifying a structure’s biological relevance

We have studied, through the score *S*, the correspondence between a multiple alignment's DC scores and the pairwise distances implied by the structure for a particular sequence in the alignment. However, to calculate *S*, there are typically many structures to choose among, and these may differ in important particulars. Recent studies [27–32] have demonstrated that high DC scoring pairs that are distant in certain benchmark 3D structures may come into contact within alternative conformations or across homo-oligomer interfaces, and have thereby provided insight into protein biophysical and dynamic properties. Other studies [33, 34] have combined DCA with correlation analyses involving larger groups of structurally and/or functionally correlated residues, thereby generating further insight. Here we illustrate the application of our method to these sorts of studies.

To the degree to which DC scores capture the pairwise correlations imposed by the functional requirements common to a protein family, we expect the *S* yielded by a particular structure to reflect the degree to which that structure exhibits critical interactions characteristic of the family. In other words, *S* may measure the degree to which a specific structural conformation is biologically relevant. To investigate this, we consider three cases—human Ran GTPase, Gna1 *N*-acetyltransferase from *C*. *elegans*, and the bacterial (*E*. *coli*) clamp loader complex. Using available structures for each of these, we add hydrogen atoms using the Reduce program [21] to better discriminate among residue-to-residue contact distances. A previous DCA analysis [31] found that the heavy atom distance distribution for directly coupled residue pairs exhibited local maxima at 2.8 Å and 3.7 Å, which were interpreted as corresponding to the *donor-acceptor* distance of hydrogen bonds and to hydrophobic interactions, respectively. Here we choose to focus on hydrogen bond interactions. Since our analyses explicitly model hydrogen atoms, we calculate *S* using a maximum structural distance of 2.6 Å, which, based on the sum of the van der Waals radii for hydrogen plus either nitrogen or oxygen [35], corresponds to an upper bound on the *hydrogen-acceptor* distance of hydrogen bonds.

#### Ran GTPase

Ran GTPase is required for the translocation of proteins and RNA through the nuclear pore complex. Ran exists in both GTP- and GDP-bound forms. Ran-mediated hydrolysis of GTP to GDP, which is believed to drive transport of cargo from the nucleus into the cytoplasm, involves the combined action of Ran GTPase activating protein (RanGAP), which activates Ran’s intrinsic GTPase activity, and of the Ran-binding proteins RanBP1 [36]. The nucleotide exchange factor RCC1 converts Ran-GDP back into Ran-GTP.

**Fig 4:**
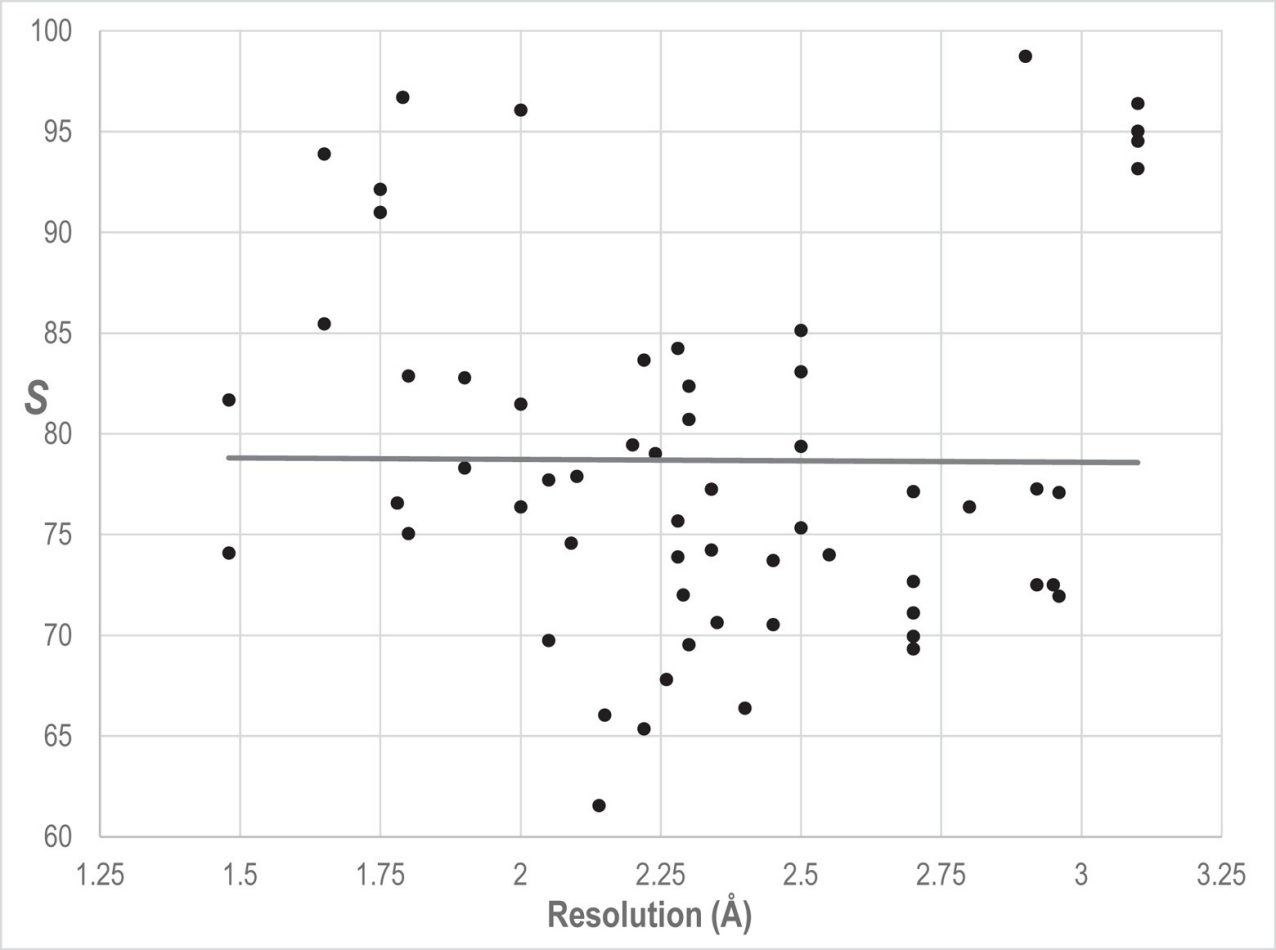
Regression analysis of *S* for 60 Ran GTPase structures versus their crystal structure resolutions. The coefficient of determination is *R*^2^ = 0.00005, indicating that crystal structure resolution fails to explain the variability of *S* around its mean. The same R^4^ family MSA and parameters were used here as for the analyses in Table 4.

**Fig 5:**
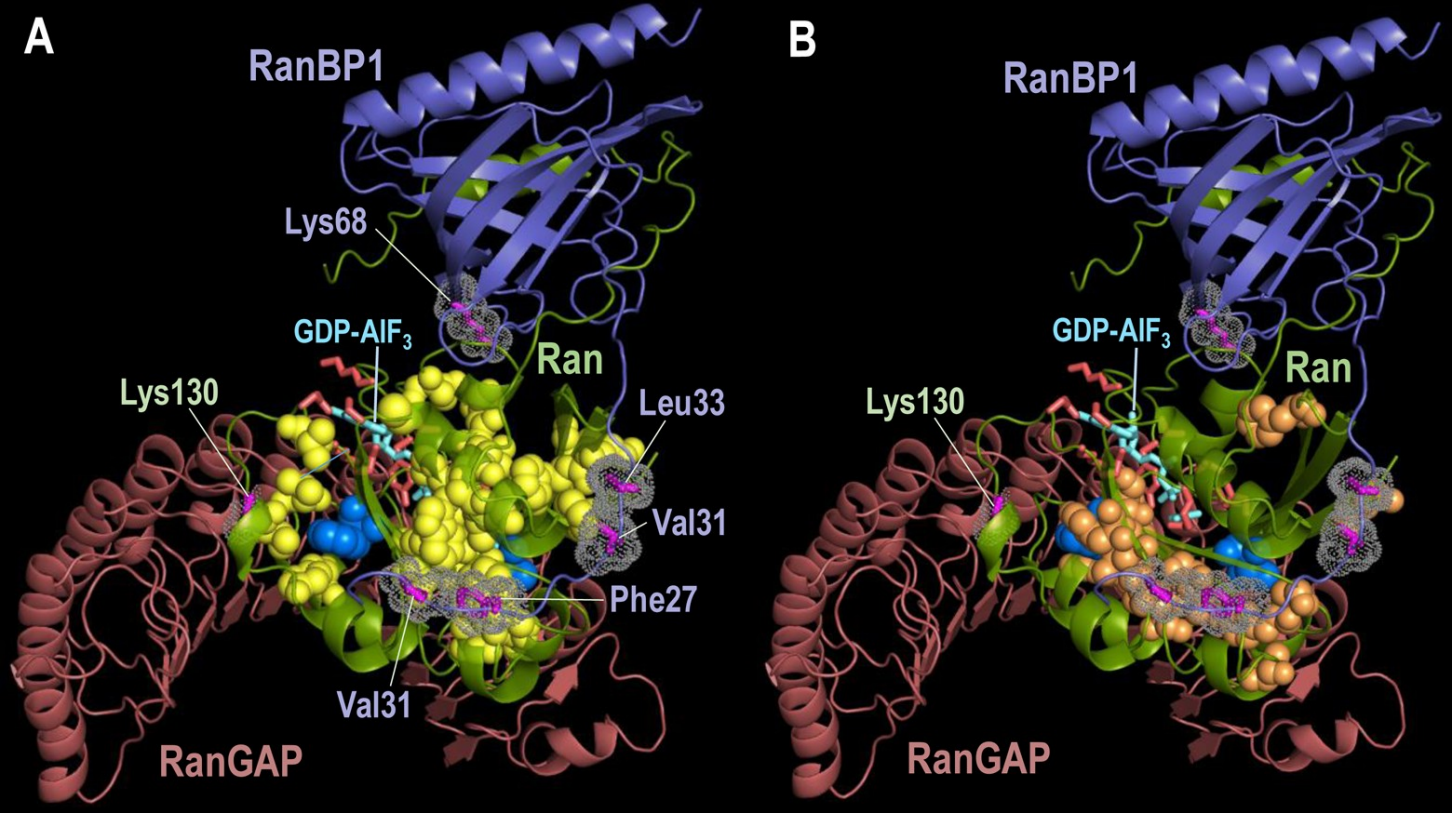
Residues in Ran involved in interacting pairs within the transition state structure (pdb: 1k5g) [37]. Sidechains of residues in RanBP1 contacting Ran are labeled in (A) and shown in magenta with dot clouds. The sidechain of Ran Lys130, which plays a role in the stimulation of GTP hydrolysis by RanGAP [37], is indicated. The GTP transition state analog and sidechains of Ran’s catalytic (active site) residues are represented as cyan and red sticks, respectively. A PyMOL session file corresponding to this figure is available at our website. **A**. Sidechains of residue pairs contributing to the higher *S* for Ran in the transition state than in the ground state (pdb: 1k5d). These residues are represented as yellow spheres, except for the pivot point residues Phe90 and Val14, which are shown as bright blue spheres, and for two of the unlabeled catalytic residues shown in red (Thr24 and Thr42). **B**. Ran residues forming pairs whose interactions remain stable over diverse conformational forms (shown as orange and bright blue spheres). These diverse forms include the Ran-RanBP1-RanGAP transition (pdb: 1k5g) and ground (pdb: 1k5d) states; Ran bound to its exchange factor, RCC1 (pdb: 1i2m); Ran bound to GDP (pdb: 3gj0); Ran bound to Ntf1 and GDP (pdb: 1a2k); and Ran bound to RanBP1 and CRM1 (pdb: 4hb2).

Two crystal structures of the Ran-RanBP1-RanGAP ternary complex are available [37]: one in the ground state (i.e., bound to a non-hydrolysable GTP analog) and another in a transition-state mimic. For each crystal structure, the unit cell contains four tertiary complexes whose Ran subunits are labeled as chains A, D, J and G. Each chain yields an *S* for each of the two structures, as shown in **Table 4**, and, on average, the *S* for the transition-state exceeds that for the ground state by 24 based on the R^4^ family MSA described below. (Note that, for Ran, we find no correspondence between *S* and crystal structure resolution, as shown in **Fig 4**.) This average difference in *S*, corresponding to greater than 24 orders of magnitude in *P*, indicates that the transition state has more functionally relevant interactions than does the ground state. A detailed investigation of the transition state interactions absent from the ground state may provide insight into this key step in Ran-mediated nuclear transport. We investigate this possibility in **Fig 5A** by showing those residues participating in pairs that, for all four Ran subunits within the crystal structure unit cell: (1) are among the left-distinguished pairs for the transition state, but not for the ground state; and (2) are closer by at least ⅓ Å in the transition state than in the ground state. These residues appear to form allosteric pathways between Ran’s active site and its sites of interaction with RanBP1 and with RanGAP. The latter site includes a salt bridge, between Lys130 of Ran and Asp225 of RanGAP, that contributes to the stimulation of GTP hydrolysis by RanGAP [37]. In contrast, residues that participate in pairwise interactions that are relatively stable among diverse conformational forms occur in regions adjacent to these putative pathways (**Fig 5B**). Notably, Phe90, which forms a stabilizing interaction with Gly121 in the guanine binding loop [38], and Val14 are the only residues that participate (based on our criteria) in both transition-state-specific and stable interactions, and therefore may function as pivot points. This analysis illustrates how one may use our approach to investigate structural changes of potential functional relevance, thereby aiding experimental studies regarding catalytic mechanisms, substrate recognition, allostery, drug design, and protein engineering.

**Table 4:** *S* for Ran GTPase in the transition state complex^a^ and in the corresponding ground state complex^b^.

Input MSA:	number of	transition state *S*^c^					ground state *S*					Δ*S*
	seqs	A^d^	D	J	G	avg	A	D	J	G	avg	avg
GTPase superfamily	274,681	53.9	53.7	54.9	53.9	54.1	42.7	42.2	45.1	41.5	42.9	11.2
R^4^ family^e^	27,571	95.0	96.4	94.5	93.2	94.8	70.0	71.1	72.7	69.3	70.8	24.0
Ran subfamily	507	5.4	5.3	5.3	5.6	5.4	6.9	6.9	6.9	6.9	6.9	-1.5

^a^Ran-GDP-AlFx-RanBP1-RanGAP; pdb: 1k5g; 3.1 Å.

^b^Ran-GppNHp-RanBP1-RanGAP; pdb: 1k5d; 2.7 Å.

^c^The *s*-scores are based on CCMpred DC scores with *L* = 12,090, on *m* = 5, and on *r* = 2.6 Å.

^d^The letters A, D, J and G correspond to the chain designations for each of four Ran subunits within the crystal structure unit cell.

^e^The R^4^ family is composed of multiple subfamilies; this includes Rab, Rho, Ras and Ran GTPases.

#### Higher *S* for a Ran subgroup of P-loop GTPases

To examine the dependence of *S* on the sequences included in the input MSA, we used Bayesian Partitioning with Pattern Selection (BPPS) [39] to classify the aligned sequences into three nested sets consisting of all P-loop GTPases, of Rab, Rho, Ras and Ran (termed R^4^) GTPases, and of Ran GTPases. We calculated *S* from the DC scores for each of these MSAs based on the Ran subunit of the Ran-RanBP1-RanGAP transition and ground state structures (**Table 4**). On average, *S* based on the R^4^ family exceeded *S* based on the GTPase superfamily by 41 and 28 for the transition and ground states, respectively. This suggests that proteins within the R^4^ subgroup share pairwise constraints and mechanistic similarities that other P-loop GTPases lack.

#### Complementarity of DCA and BPPS analyses of Ran

Like DCA, BPPS identifies correlations among MSA columns, but unlike DCA it focuses on detecting family-specific sequence patterns associated with functional specialization rather than on pairwise correlations. There can be some overlap between the patterns of correlation detected by the two approaches, but such overlap is typically fairly weak. For illustrative purposes, we consider the DCA array used for the analysis of the R^4^ family in Table 4. As shown in **S1A Fig**, when pairs of positions separated by ≤ 5 Å are distinguished, the optimal initial cluster, highlighted in yellow, is highly significant (*S* = 230); 64% of the pairs in this cluster are distinguished, and 54% of all distinguished pairs are in the cluster. These high percentages reflect DCA's success in detecting directly interacting residues. BPPS defines the R^4^ family by recognizing positions having distinctive residue patterns, and, for comparison to S1A Fig, we distinguish in **S1B Fig** the elements of the DCA array corresponding to pairs of these positions. Again there is a significant (*S* = 6.2) initial cluster, highlighted in yellow. However, only 6% of the pairs in this cluster are distinguished, and only 16% of all the distinguished pairs are in the cluster. Thus, while there is a weak tendency for pairs of positions recognized by BPPS as characterizing the R^4^ family to receive high DC scores, a sizable majority of these pairs do not. In general, DCA and BPPS often recognize correlations of a complementary character. BPPS, in focusing on positions whose residue patterns are distinctive of a particular family, often recognizes correlations among positions on the protein’s surface or far removed spatially, and whose interaction is not direct but rather linked through common function [39].

#### Homodimeric Gna1 *N*-acetyltransferase

For the preceding analysis, we examined spatial contacts only within single protein subunits, whereas correlated mutations are also associated with contacts at homo-oligomer interfaces. To consider such contacts as well, we applied our approach to the homodimeric structure of glucosamine-6-phosphate *N*-acetyltransferase (Gna1) [40], a GCN5-like *N*-acetyltransferase (GNAT) [41] that transfers an acetyl group from coenzyme A (CoA) to glucosamine-6-phosphate to produce *N*-acetyl-D-glucosamine-6-phosphate (GlcNAc-6P). (In a previous study [42], we found that the residues most characteristic of the GNAT family to which Gna1 belongs are contributed by both subunits to form the active site at the homodimeric interface. This contrasted with the GNAT superfamily’s most characteristic residues, which are remote from the homodimeric interface.) To study the influence on *S* of including homodimeric interface contacts, either in Gna1 bound to CoA or in Gna1 bound to both CoA and the reaction product GlcNAc-6P, we computed pairwise distances based either solely on contacts internal to each subunit or on both internal and interface contacts. In the latter case, we used the shorter of the two contact distances to rank each residue pair. The inclusion of trans-homodimer contacts significantly increased *S* both for the product-bound complex (Δ*S* = 11.5 and 13.1), and for the unbound complex (Δ*S* = 8.7 and 11.6) (**Table 5**). This suggests that homodimerization plays an important role in substrate binding or catalysis or both. Note that binding of product yielded little or no increase in *S* based either on internal contacts only or on internal plus trans-homodimer contacts.

**Table 5:** *S* as a measure of biological relevance for the *N*-acetyltransferase Gna1 bound either to coenzyme A (CoA) (pdb: 4ag7; 1.55 Å) or to both CoA and *N*-acetyl-D-glucosamine-6-phosphate (GlcNAc-6P) (pdb: 4ag9; 1.76 Å).

structural state		distance-based *S*					
		A^a^	A:B^b^	Δ*S*	B^a^	B:A^b^	Δ*S*
Gna1 + CoA		48.6	57.3	8.7	46.5	58.1	11.6
Gna1 + CoA + GlcNAc-6P		43.4	54.9	11.5	45.0	58.1	13.1
	Δ*S*	-5.2	2.4		-1.5	0.0	

The *s*-scores are based on CCMpred DC scores, using the corresponding MSA from Table 2, on *r* = 2.6 Å and on *m* = 5.

^a^The letters A and B correspond to the chain designations for the individual subunits; *S* in these columns is based solely on internal contacts.

^b^*S* in these columns is based on internal and homodimeric contacts; the letter to the right of the colon represents the subunit from which trans-homodimer pairwise distances were obtained.

Because the homodimeric interface includes many pattern residues characteristic of the Gna1 family [42], we also considered to what extent the DCA and BPPS analyses are complementary (**Table 6**). Unlike for Ran GTPase, the highest DC scoring residue pairs correspond, with high significance (*S* = 27.9), to pairs of the 25 highest BPPS-scoring residues characteristic of the Gna1-family. Thus, the degree of complementarity between DCA and BPPS is protein-specific. Note, however, that the overlap between Gna1-family BPPS pairs and either DCA or 3D contacting pairs is far from optimal (**S2 Fig**), suggesting that, in this case as well, pairs of the highest BPPS-scoring residues are fairly distinct from residue pairs with the highest DC scores and the shortest 3D distances.

**Table 6:** *S* as a measure of the overlap between pairs of BPPS pattern residues and the highest DC scoring pairs for the *N*-acetyltransferase Gna1 bound to both CoA and *N*-acetyl-D-glucosamine-6-phosphate (GlcNAc-6P) (pdb: 4ag9; 1.76 Å).

characteristic pattern		pattern-based *S*	pattern residue positions^a^
GNAT superfamily		2.2	153,116,83,118,154,103,31,121,113,24,106,82, 108,117,145,149,137,25,148,73,36,122,79
Gna1 family		27.9	135,90,92,104,68,95,93,44,141,136,43,102,105, 134,40,58,36,101,98,94,35,116,89,54,61
	Δ*S*	25.7	

^a^Pattern residues were determined in [39, 42]; positions are ordered by decreasing BPPS score.

#### DNA clamp loader complex

To further explore the possible relationship between a structure’s biological relevance and its score *S*, we examined subunits of the bacterial DNA clamp loader complex. This complex forms a spiral-shaped semicircle of two inactive subunits, δ and δ’, and three γ ATPase subunits arranged in the order: δ-γ-γ-γ-δ’. The last two γs and δ’ each functionally interact with the ATP-binding site of the preceding γ subunit. This complex loads a sliding clamp onto primer template DNA. The ψ protein binds to the clamp loader, thereby coupling it to single-stranded DNA-binding protein. Upon binding to DNA and ATP, ψ promotes the clamp-loading activity of the complex by stabilizing it in a spiral-shaped conformation consistent with recognition of both RNA and DNA primers [43].

We analyzed two different clamp loader structures: one of the unbound clamp loader complex (pdb_id: 1jr3) [44] and another of the clamp loader bound to primer template DNA and to the ψ protein and with an analog of ATP bound to each of the γ subunits (pdb_id: 3gli) [43]. First, using jackhmmer [45], we created one MSA for each of the subunits: δ, γ and δ’, and used CCMpred to generate an ordered DCA array from each MSA. Second, we calculated values of *S* for each array using corresponding structures for the δ, γ and δ’ subunits (**Table 7**). Note that in the bound form, there are two clamp loader complexes in the unit cell of the crystal structure, yielding two distinct structures for each of the five subunits. The difference between the *s*-scores for the bound and unbound forms, Δ*S*, ranges from 3 to 58 with a mean of 26, which is highly significant. This conforms to the expectation that the biologically more relevant bound conformation will yield higher *S* than the unbound form, and further illustrates how *S* can be used to evaluate a structure’s biological relevance. However, unlike for Gna1, the inclusion of contacts between adjacent γ subunits decreases *S*, suggesting that, in this case, homo-oligomer interactions fail to impose detectable constraints. Finally, we explored for clamp loader subunits the putative contributions to direct couplings of hydrogen bond interactions (pairwise distances ≤ 2.6 Å; Table 7) versus hydrophobic interactions (pairwise distances ≥ 3 Å and ≤ 5 Å; **Table 8**). This comparison suggests that the biologically relevant clamp loader state favors presumably more geometrically specific hydrogen bond interactions over presumably less specific hydrophobic interactions.

**Table 7:** *S* as a measure of structural biological relevance for the bacterial DNA clamp loader complex based on a maximum distance of 2.6 Å.

subunit	# aligned	*L*	Unbound^a^	bound to ψ + “ATP” + DNA^b^					
	seqs		*S*	*S*	Δ*S*	*S*	Δ*S*	*S*-adjacent^c^	
**δ**-γ-γ-γ-δ’	8,765	47,914	135.4	153.8	18	157.5	22	n.a.	n.a.
δ-**γ**-γ-γ-δ’	24,739	43,694	148.0	172.1	24	172.6	25	168.3	165.5
δ-γ-**γ**-γ-δ’	″	″	146.5	162.7	16	167.9	21	156.0	158.4
δ-γ-γ-**γ**-δ’	″	″	154.3	157.5	3	169.0	15	155.1	164.9
δ-γ-γ-γ-**δ’**	23,512	32,439	65.2	122.8	58	118.9	54	n.a.	n.a.

These analyses are based on CCMpred DC scores with *m* = 5.

^a^Based on a 2.7 Å structure (pdb: 1jr3), for which the subunits δ, γ_1_, γ_2_, γ_3_ and δ’ are labeled as chains D, C, A, B and E, respectively.

^b^Based on a 3.5 Å structure (pdb: 3gli) that contains τ instead of γ, which is a shorter variant of τ. The two *S* and two Δ*S* columns correspond to two clamp loader complexes within the crystal structure unit cell. For the first complex the subunits δ, γ_1_, γ_2_, γ_3_ and δ’ are labeled as chains A, B, C, D and E, respectively, and for the second complex as chains F, G, H, I and J, respectively.

^c^*S* based on contacts both internal to each γ and with adjacent γ subunit(s); n.a. = not applicable.

**Table 8:** *S* as a measure of structural biological relevance for the bacterial DNA clamp loader complex based on a minimum distance of 3 Å and a maximum of 5 Å.

subunit	# aligned	Unbound^a^		bound to ψ + “ATP” + DNA^b^					
	seqs	*L*	*S*	*L*	*S*	Δ*S*	*L*	*S*	Δ*S*
**δ**-γ-γ-γ-δ’	8,765	47,669	120.4	47,647	107.9	-12.5	47,653	110.4	-10.0
δ-**γ**-γ-γ-δ’	24,739	43,447	160.1	43,428	128.4	-31.7	43,429	130.5	-29.6
δ-γ-**γ**-γ-δ’	″	43,440	151.5	43,432	130.4	-21.1	43,433	136.6	-14.9
δ-γ-γ-**γ**-δ’	″	43,438	127.6	43,436	135.5	7.8	43,442	145.4	17.8
δ-γ-γ-γ-**δ’**	23,512	32,240	109.7	32,198	91.0	-18.7	32,201	92.1	-17.6

This analysis is based on CCMpred DC scores with *m* = 5, and focuses on putative hydrophobic interactions as opposed to the focus in Table 7 on putative hydrogen bond interactions. Note that, for these, *L* decreases slightly by the number of pairs less than 3 Å apart for each structure, therefore each *S* is based on a different value of *L*

^a^See footnote a in Table 7.

^b^See footnote b in Table 7.

## Discussion

STARC-computed *s*-scores quantify a DCA method’s ability to detect 3D residue-to-residue contacts. When used in combination with the Wilcoxon signed rank test, they yield a significance measure of the performance of one method versus another and can quantify, as well, a method’s tendency to detect indirect couplings. Larger domains tend to yield higher *s*-scores due to enhanced statistical power. However, this is not a confounding factor when *s*-scores are used to compare different DCA methods applied to the same MSA and 3D structure or to compare alternative structures based on the same DCA method and MSA.

The following advantages of *S*-scores over *S*_*F*_-scores and PPV may be noted: *S*-scores are not biased toward any particular method, but rather correspond to the optimal value of *F* for each method and reference structure under consideration; this may reveal important features that would otherwise be overlooked. *S*-scores avoid ranking inconsistencies due to one’s choice of fixed values of *F*. *S*-scores can tap into additional information regarding each structure’s possible biological relevance, as illustrated here. *S*-scores take into consideration not only the number and arrangement of (false negative) contacting residue pairs to the right of a potential cut point, but also the ordering of those pairs based on their 3D distances within a reference structure. And, unlike PPV, *S*-scores provide a measure of statistical significance. Of course, researchers also have the option of computing *S*_*F*_, thereby obtaining both a measure of significance and an assessment of program performance as functions of *F*.

We could further develop the STARC statistical model by considering the arrangement of the *d* distinguished pairs before *X*. A pair with a higher DC score should be more likely than one with a lower DC score to correspond to a 3D interaction. Ideally, the *d* pairs should thus be arranged in order of decreasing DC score. To measure how closely a DCA method’s output comes to achieving this configuration, we may first define a permutation π by ranking the *d* distinguished pairs based on 3D distance, with smaller distances receiving superior ranks (i.e., lower numbers), and then define $\tau \left(\pi \right)=\frac{1}{2}\sum_{i=1}^{d}{\left(\pi \left[i\right]-i\right)}^{2}$. One may show that τ is an integer function that for random permutations is symmetrically distributed about its mean *μ* = (*d*^3^−*d*)/12, with standard deviation $\sigma =\mu /\sqrt{d-1}$. For *d* ≤ 16 one may compile exact *p*-values for τ by exhaustive enumeration, and for *d* > 16 estimate them using either a Gaussian approximation or Monte Carlo simulation. However, it is unclear whether these *p*-values are independent of *P*_*a*_ and *P*_*b*_, and whether there is biological benefit to including this order in our statistical model. We plan to investigate these questions.

An important potential application of our approach, which is beyond the scope of this study, is the evaluation of MSA accuracy without the need for benchmark alignments, which typically contain a relatively small number of sequences and whose accuracy may be uncertain [46]. Our proposed approach would proceed on the assumption that, given available structures, more accurate MSAs will yield higher values of *S*. We are developing this approach, which should benefit from the large amount of sequence and structural data becoming available.

Our analysis of Ran, Gna1 and the DNA clamp loader complex suggests that *S* may be useful for evaluating the biological relevance of alternative structural conformations of the same protein and for characterizing the nature of conformation-specific interactions. Viewing direct couplings as functionally imposed constraints and proteins as molecular machines, *S* may measure the degree to which a particular crystal structure captures a protein in a mechanistically important state. If so, then analyzing in what ways various residue pairs contribution to *S* may provide mechanistic clues. Likewise, comparative analyses among MSAs corresponding to a protein’s subfamily, family and superfamily may provide mechanistic clues regarding functional specialization. Our analysis here also suggests that one may use STARC to search for the most biologically relevant among the many structures often available for a major protein superfamily.

## Methods

### Protein structural coordinates

For the thirty STARC analyses in Table 2, we obtained high quality crystal structures from the Protein Data Bank (PDB) (www.rcsb.org/pdb). The pdb and chain identifiers are given in column 1 of Table 2. Likewise, the coordinates for the Ran, Gna1 and DNA clamp loader analyses were obtained from the PDB; their pdb identifiers are given in Tables 4, 5 and 6, respectively. For all analyses, hydrogen atoms were added using the Reduce program [31], except for the pdb coordinate file for 3f1lA in which hydrogens were already present. Hence, residue-to-residue distances are based on any two atoms, including hydrogens, albeit ignoring main chain to main chain interactions. This allows better discrimination among hydrogen bond interactions based on subtle differences in contact distances.

### DCA methods

EVcouplings (EVC) was run over the EVfold website (http://evfold.org) using the pseudo-likelihood maximization (PLM) option with default settings. For each analysis, taking as input the sequence corresponding to the reference structure as the query, EVcouplings uses jackhmmer [45] to create an MSA, from which it then computes the DC scores. The score file and the corresponding PDB coordinates serve as the input to STARC. We also used the jackhmmer alignment as input to the other programs. The GaussDCA program was run with Frobenius norm ranking (with default parameters); this was done interactively under Julia (www.julialang.org). PSICOV version 2.4 was run using the author recommended –p and –d 0.03 options and the jackhmmer alignment reformatted by the fasta2aln program, which is included with the PSICOV package (http://bioinf.cs.ucl.ac.uk/downloads/PSICOV). CCMpred version 0.3.2 (https://travis-ci.org/soedinglab/CCMpred) was run with default settings again using the reformatted alignment. Note that the output from GaussDCA, CCMpred and PSICOV does not include the query sequence, which, along with the DC scores, were provided as input to STARC.

### Simulations

We performed two types of simulations for each of the six MSAs in Fig 1, which are labeled by their corresponding pdb identifiers, 3fhkF, 1ijxA, 4cmlA, 1k30A, 1olzA, 1wznA, and which correspond to analyses in Table 2. These MSAs vary substantially in their numbers of aligned columns (127 to 481) and aligned sequences (1,042 to 146,217), and in the degree of shared sequence similarity. For the first type of simulation, we randomly shuffled the DC score array for each of 100,000 runs. This simulation corresponds to the theory behind the ICA algorithm, which is described in the next section. The second type of simulation corresponds more closely to a STARC analysis by computing a DC score array from a simulated MSA. For each MSA, we first randomly permuted the residues in each aligned column and then randomly permuted the order of the columns in the MSA (termed a column-permuted MSA). Next, using these simulated MSAs as input to the CCMpred program [10], DC scores were computed for each of 100,000 runs. Finally, for each run, STARC was applied using as input the DC scores and the corresponding protein structure.

### Initial Cluster Analysis

We describe Initial Cluster Analysis (ICA) in detail elsewhere [15], but summarize the approach briefly here. ICA seeks to determine whether a set of distinguished elements within a linear array is clustered significantly near the start of the array and, if so, what is the most significant initial cluster of these elements. Abstractly, given a linear array of length *L* containing *D* '1's (the distinguished elements) and *L*-*D* '0's, it considers a generative model in which the '1's occur with particular and differing probabilities before and after a cut point *X* in the array. For any particular *X* it is relatively easy to calculate a likelihood L(X) of the array of data, and one may optimize L(X) by simply evaluating it for all possible *X*. However, the values of L(X) for close values of *X* are highly correlated, dependent upon a calculable "density of independent trials" ρ(*X*). Because ρ(*X*) is not constant but rather grows approximately as the reciprocal of *X*'s distance from 0 or *L*, simply optimizing L(X) inherently favors, *a priori*, small or large values of *X*. Therefore, if one's application suggests no such bias, choosing to optimize L(X)/ρ(X) rather than L(X) for a given array of '0's and '1's may be a better strategy. This is referred to in [15] as using "flattened priors", and is the approach we take here. ICA estimates the effective total number of independent trials implicit in either optimization, which it uses in calculating a *p*-value for the optimal *X* from its corresponding *P*_*a*_. This provides a mathematically principled way to define an optimal initial cluster of distinguished elements, balancing the claims of very short and dense clusters with those of longer but sparser clusters.

We have extended ICA here by taking account not only of distinguished elements within an array, but of a ranking assigned to these elements as well. Thus we seek here initial clusters not only with a high density of distinguished elements, but clusters in which these elements have relatively better rankings. Our *s*-score may be understood as providing a measure of the congruence between two orderings, as well as, simultaneously, an assessment of statistical significance.

### Wilcoxon signed rank test

We evaluated the performance of alternative DCA methods using the Wilcoxon signed-rank test [22], first dividing each *S* by the total number of residue pairs *L*. For CCMpred, EV-couplings and GaussDCA, these normalized *s*-scores then approximately follow a Gaussian distribution, as indicated by the Shapiro-Wilk test statistic [47] (p = 0.52, 0.60, and 0.09, respectively). For PSICOV the Shapiro-Wilk test score corresponded to *p* = 0.04, which is slightly below the acceptance threshold of *p* > 0.05.

### The STARC algorithm

The STARC program uses a modified version of the Initial Cluster Analysis (ICA) algorithm [15] to find the optimal score *S*, as described above. Alternatively, as an option, it will calculate *S*_*F*_ given a specified *F*. STARC converts PSICOV and GaussDCA formatted DC score files into EVcouplings format automatically; this requires as input the query sequence in fasta2aln format. We modified the CCMpred source code and recompiled the program to generate PSICOV-formatted DC score files. The source code for STARC is freely available at: http://evaldca.igs.umaryland.edu/.

### BPPS analysis

BPPS [39, 48] partitions the sequences in a superfamily MSA into families and subfamilies. It uses Markov chain Monte Carlo (MCMC) sampling to stochastically move sequences between subgroups, while modifying each subgroup’s characteristic pattern. BPPS also identifies and removes unrelated or aberrant sequences. We applied BPPS here to generate both family and subfamily MSAs for sequences of interest. Here we also use STARC to assess the correspondence between pairs of BPPS-defined pattern residues and high DC-scoring pairs.

## Supporting information

S1 Table Protein structural coordinates used for the Ran GTPase analysis in Fig 4. (PDF)

S1 Fig Distinguished residue pairs within an array of length *L* = 12,090, ordered by DC scores for Ran GTPase (pdb: 1k5g), as computed by CCMpred using the R^4^ MSA (see Table 4). Distinguished pairs are represented by black and red blocks, the latter indicating pairs common to panels A and B; the remaining pairs are represented by dots. The region up to each cut point *X* is highlighted in yellow. **A**. Distinguished elements are those pairs separated by ≤ 5 Å in chain A of 1k5g. ICA results: *S* = 230; *D* = 346; *X* = 291; *d* = 186; 54% of the distinguished pairs (*d*/*D*) occur in the initial 2.4% of the array (*X* /*L*). **B**. Distinguished elements are pairs of the 25 residues found by the BPPS program to be most distinctive of R^4^ GTPases. ICA results: *S* = 6.2; *D* = 281; *X* = 772; *d* = 46; 16% of the distinguished pairs occur in the initial 6.4% of the array. Note that because no ranking is available for the distinguished pairs in panel B we calculate *S* for both panels without the ball-in-urn component *P*_*b*_ and using only *P*_*a*_ [14].
(PDF)

S2 Fig Distinguished residue pairs within an array of length *L* = 8,534, ordered by DC scores for Gna1 (pdb: 4ag9), as computed by CCMpred using the MSA for the corresponding analysis in Table 2. Distinguished pairs are represented by black and red blocks, the latter indicating pairs common to panels A and B; the remaining pairs are represented by dots. The region up to each cut point *X* is highlighted in yellow. **A**. Distinguished elements are those pairs in 4ag9 separated by ≤ 5 Å within chain A or between chains A and B (whichever is shorter). ICA results: *S* = 91; *D* = 260; *X* = 663; *d* = 144; 55% of the distinguished pairs (*d*/*D*) occur in the initial 7.8% of the array (*X* /*L*). **B**. Distinguished elements are pairs of the 25 residues found by the BPPS program to be most distinctive of the Gna1 family. ICA results: *S* = 27.9; *D* = 263; *X* = 1,238; *d* = 114; 43% of the distinguished pairs occur in the initial 14.5% of the array. Note that because no ranking is available for the distinguished pairs in panel B we calculate *S* for both panels without the ball-in-urn component *P*_*b*_ and using only *P*_*a*_ [14].
(PDF)

S1 Dataset Excel file containing the *S*-score data for the analyses in Table 2. (XLSX)

S2 Dataset Excel file containing the *S*_*F*_-score data for the analyses in Table 2. (XLSX)
