# Supplementary material for: Statistical investigations of protein residue direct couplings
Source: PLoS Comput Biol. 2018 Dec 31;14(12):e1006237. doi: 10.1371/journal.pcbi.1006237 (PMC6329532; doi:10.1371/journal.pcbi.1006237)
Supplement: S1 Table — (PDF) [file pcbi.1006237.s001.pdf]

S1 Table. Protein structural coordinates used for the Ran GTPase analysis in Fig 4.

PDB	*S*	Description	Resol. (Å)
**1A2KC**	79	Gdpran-Ntf2 Complex	2.5
1A2KD	83	Gdpran-Ntf2 Complex	2.5
1A2KE	85	Gdpran-Ntf2 Complex	2.5
1IBRA	82	Complex Of Ran With Importin Beta	2.3
1IBRC	81	Complex Of Ran With Importin Beta	2.3
**1K5DA**	70	Ran-Gppnhp-Ranbp1-Rangap Complex	2.7
**1K5DD**	71	Ran-Gppnhp-Ranbp1-Rangap Complex	2.7
**1K5DG**	69	Ran-Gppnhp-Ranbp1-Rangap Complex	2.7
**1K5DJ**	73	Ran-Gppnhp-Ranbp1-Rangap Complex	2.7
**1K5GA**	95	Ran-Gdp-Alfx-Ranbp1-Rangap Complex	3.1
**1K5GD**	96	Ran-Gdp-Alfx-Ranbp1-Rangap Complex	3.1
**1K5GG**	93	Ran-Gdp-Alfx-Ranbp1-Rangap Complex	3.1
**1K5GJ**	95	Ran-Gdp-Alfx-Ranbp1-Rangap Complex	3.1
3A6PC	73	Exportin-5:rangtp:pre-Mirna Complex	2.92
3A6PH	77	Exportin-5:rangtp:pre-Mirna Complex	2.92
4GPTA	84	Kpt251 With Crm1-ran-ranbp1	2.22
4GMXA	78	Kpt185 With Crm1-Ran-Ranbp1	2.1
4HATA	77	Crm1 Inhibitor Leptomycin B With Crm1- Ran-ranbp1	1.78
4HAUA	96	Crm1 Inhibitor Ratjadone A With Crm1- Ran-ranbp1	2
4HAVA	76	Crm1 Inhibitor Anguinomycin A With Crm1-ran-ranbp1	2
4HAWA	78	Crm1 Inhibitor Leptomycin B With Crm1(K548A)-ran-ranbp1	1.9
4HAXA	84	Crm1 Inhibitor Ratjadone A With Crm1(K579A)-ran-ranbp1	2.28
4HAYA	70	Crm1 Inhibitor Leptomycin B With Crm1(K548E,K579Q)-ran-ranbp1	2.3
4HAZA	83	Crm1 Inhibitor Leptomycin B With Crm1(R543S,K548E,K579Q)-ran-ranbp1	1.9
4HB0A	79	Crm1 Inhibitor Leptomycin B With Crm1(K541Q,K542Q,R543S,K545Q,K548Q,K579Q)-ran-ranbp1	2.2
**4HB2A**	83	Crm1-ran-ranbp1	1.8
4HB3A	76	Crm1(t539s)-ran-ranbp1 With Weakly Bound Unmodeled Leptomycin B	2.8
4HB4A	78	Crm1 Inhibitor Leptomycin B With Crm1(537dltvk541/glceq)-ran-ranbp1	2.05
4OL0A	99	Transportin-sr2, A Karyopherin Involved In Human Disease, In Complex With Ran	2.9
4WVFA	75	Kpt276 In Complex With Crm1-ran-ranbp1	1.8
5CIQA	85	Ran Gdp Wild Type Tetragonal Crystal Form	1.65
5CIQB	94	Ran Gdp Wild Type Tetragonal Crystal Form	1.65
5CITA	91	Ran Gdp Wild Type Monoclinic Crystal Form	1.75
5CITB	92	Ran Gdp Wild Type Monoclinic Crystal Form	1.75
**3GJ0A**	82	Human Rangdp	1.48
**3GJ0B**	74	Human Rangdp	1.48
3GJ3A	97	Human Rangdp-Nup153znf2 Complex	1.79
5DH9A	74	Pki Nes Flip Mutant Peptide In Complex With Crm1- Ran-ranbp1	2.55
5DHAA	73	Cpeb4 Nes Reverse Mutant Peptide In Complex With Crm1-ran-ranbp1	2.95
5DHFA	72	Hrio2 Nes Peptide In Complex With Crm1-ran-ranbp1	2.29
5DI9A	76	Hrio2 Nes Reverse Mutant Peptide In Complex With Crm1-ran-ranbp1	2.28
5DIFA	75	Cpeb4 Nes Peptide In Complex With Crm1-ran-ranbp1	2.09
5JLJA	75	Kpt8602 In Complex With Crm1-ran-ranbp1	2.5
5UWHA	68	Paxillin Nes Peptide In Complex With Crm1-ran- Ranbp1	2.26
5UWIA	62	Hdac5 Nes Peptide In Complex With Crm1-ran-ranbp1	2.14
5UWJA	65	Fmrp Nes Peptide In Complex With Crm1-ran-ranbp1	2.22
5UWOA	71	Engineered Fmrp-1b Nes Peptide In Complex With Crm1-ran-ranbp1	2.35
5UWPA	70	Mdia2 Nes Peptide In Complex With Crm1-ran-ranbp1	2.05
5UWQA	74	Cdc7 Nes Peptide In Complex With Crm1-ran-ranbp1	2.28
5UWRA	79	Cdc7 Nes Peptide (extended) In Complex With Crm1- Ran-ranbp1	2.24
5UWSA	66	X11l2 Nes Peptide In Complex With Crm1-ran-ranbp1	2.4
5UWTA	74	Hxk2 Peptide In Complex With Crm1 K579a Mutant- Ran-ranbp1	2.34
5UWUA	77	Smad4 Nes Peptide In Complex With Crm1-ran-ranbp1	2.34
5UWWA	66	Deaf1 Peptide In Complex With Crm1 K579a Mutant- Ran-ranbp1	2.15
**1RRPA**	72	Ran-Gppnhp-Ranbd1 Complex	2.96
**1RRPC**	77	Ran-Gppnhp-Ranbd1 Complex	2.96
5CLLA	74	Truncated Ran Wild Type In Complex With Gdp-bef And Ranbd1	2.45
5CLLC	71	Truncated Ran Wild Type In Complex With Gdp-bef And Ranbd1	2.45
1WA5A	81	The Exportin Cse1p Complexed With Its Cargo (Kap60p) And Rangtp	2
3W3ZB	77	Kap121p Bound To RanGTP	2.7
