# Supplementary material for: Statistical investigations of protein residue direct couplings
Source: PLoS Comput Biol. 2018 Dec 31;14(12):e1006237. doi: 10.1371/journal.pcbi.1006237 (PMC6329532; doi:10.1371/journal.pcbi.1006237)
Supplement: S1 Fig — Distinguished pairs are represented by black and red blocks, the latter indicating pairs common to panels A and B; the remaining pairs are represented by dots. The region up to each cut point X is highlighted in yellow. A. Distinguished elements are those pairs separated by ≤ 5 Å in chain A of 1k5g. ICA results: S = 230; D = 346; X = 291; d = 186; 54% of the distinguished pairs (d/D) occur in the initial 2.4% of the array (X /L). B. Distinguished elements are pairs of the 25 residues found by the BPPS program to be most distinctive of R4 GTPases. ICA results: S = 6.2; D = 281; X = 772; d = 46; 16% of the distinguished pairs occur in the initial 6.4% of the array. Note that because no ranking is available for the distinguished pairs in panel B we calculate S for both panels without the ball-in-urn component Pb and using only Pa [14]. (PDF) [file pcbi.1006237.s002.pdf]

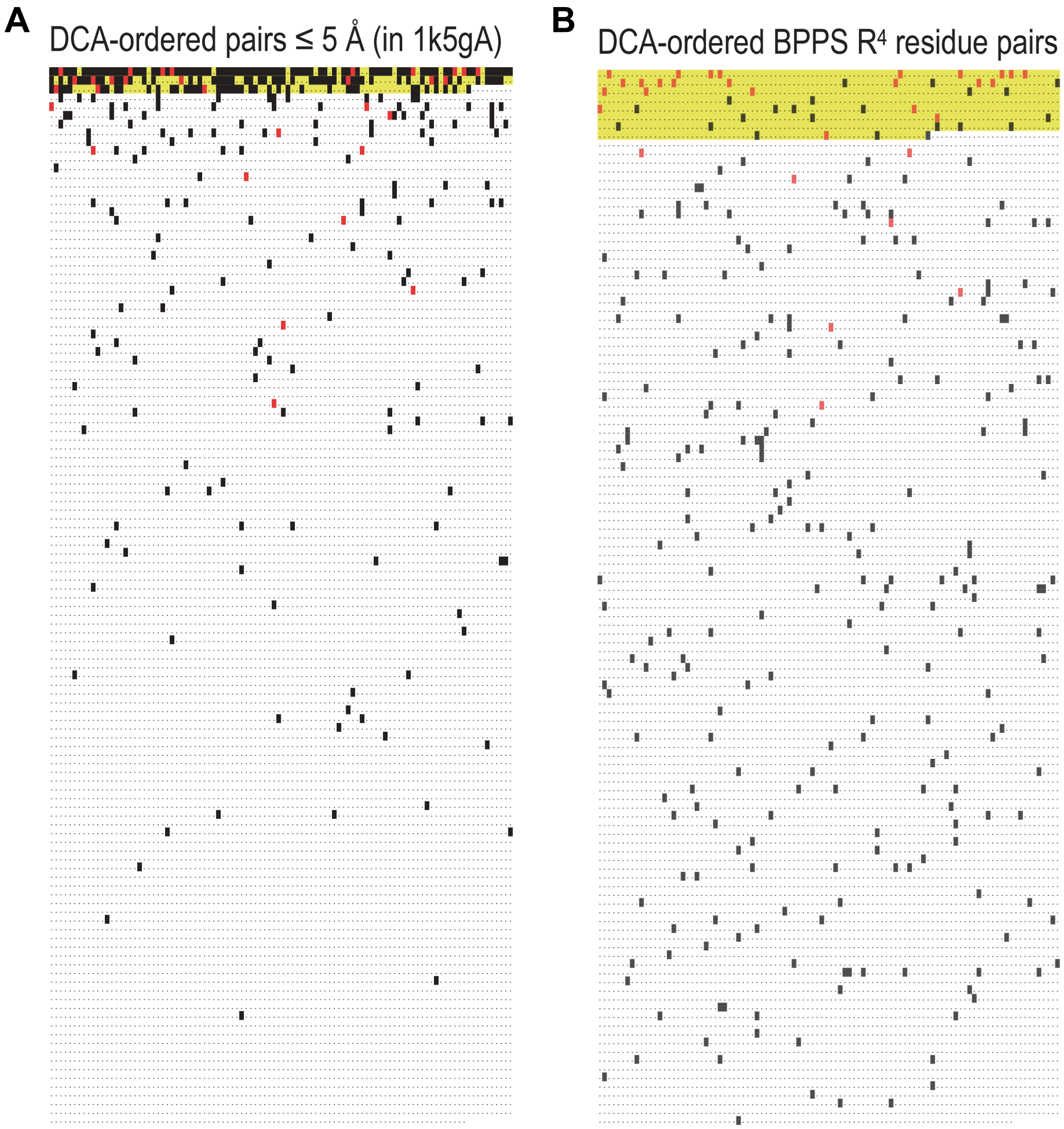

S1 Fig. Distinguished residue pairs within an array of length L = 12,090, ordered by DC scores for Ran GTPase (pdb: 1k5g), as computed by CCMpred using the R^4 MSA (see Table 4). Distinguished pairs are represented by black and red blocks, the latter indicating pairs common to panels A and B; the remaining pairs are represented by dots. The region up to each cut point X is highlighted in yellow. **A**. Distinguished elements are those pairs separated by ≤ 5 Å in chain A of 1k5g. ICA results: S = 230; D = 346; X = 291; d = 186; 54% of the distinguished pairs (d/D) occur in the initial 2.4% of the array (X/L). **B**. Distinguished elements are pairs of the 25 residues found by the BPPS program to be most distinctive of R^4 GTPases. ICA results: S = 6.2; D = 281; X = 772; d = 46; 16% of the distinguished pairs occur in the initial 6.4% of the array. Note that because no ranking is available for the distinguished pairs in panel B we calculate S for both panels without the ball-in-urn component P_b and using only P_a [14].
