# Supplementary material for: Statistical investigations of protein residue direct couplings
Source: PLoS Comput Biol. 2018 Dec 31;14(12):e1006237. doi: 10.1371/journal.pcbi.1006237 (PMC6329532; doi:10.1371/journal.pcbi.1006237)
Supplement: S2 Fig — Distinguished pairs are represented by black and red blocks, the latter indicating pairs common to panels A and B; the remaining pairs are represented by dots. The region up to each cut point X is highlighted in yellow. A. Distinguished elements are those pairs in 4ag9 separated by ≤ 5 Å within chain A or between chains A and B (whichever is shorter). ICA results: S = 91; D = 260; X = 663; d = 144; 55% of the distinguished pairs (d/D) occur in the initial 7.8% of the array (X /L). B. Distinguished elements are pairs of the 25 residues found by the BPPS program to be most distinctive of the Gna1 family. ICA results: S = 27.9; D = 263; X = 1,238; d = 114; 43% of the distinguished pairs occur in the initial 14.5% of the array. Note that because no ranking is available for the distinguished pairs in panel B we calculate S for both panels without the ball-in-urn component Pb and using only Pa [14]. (PDF) [file pcbi.1006237.s003.pdf]

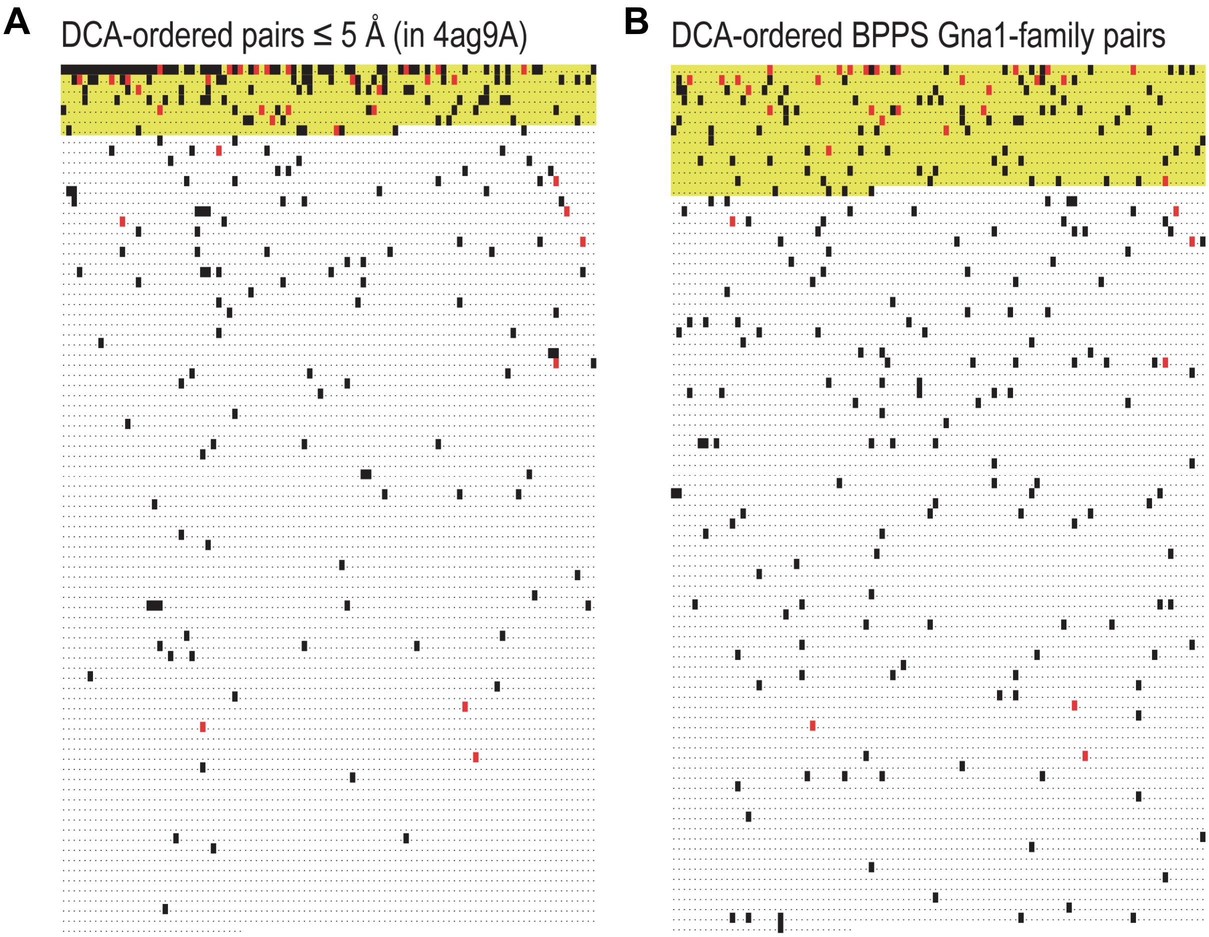

S2 Fig. Distinguished residue pairs within an array of length L = 8,534, ordered by DC scores for Gna1 (pdb: 4ag9), as computed by CCMpred using the MSA for the corresponding analysis in Table 2. Distinguished pairs are represented by black and red blocks, the latter indicating pairs common to panels A and B; the remaining pairs are represented by dots. The region up to each cut point X is highlighted in yellow. **A**. Distinguished elements are those pairs in 4ag9 separated by ≤ 5 Å within chain A or between chains A and B (whichever is shorter). ICA results: S = 91; D = 260; X = 663; d = 144; 55% of the distinguished pairs (d/D) occur in the initial 7.8% of the array (X/L). **B**. Distinguished elements are pairs of the 25 residues found by the BPPS program to be most distinctive of the Gna1 family. ICA results: S = 27.9; D = 263; X = 1,238; d = 114; 43% of the distinguished pairs occur in the initial 14.5% of the array. Note that because no ranking is available for the distinguished pairs in panel B we calculate S for both panels without the ball-in-urn component P_b and using only P_a [14].
